# Supplementary material for: Innovative GenExpA software for selecting suitable reference genes for reliable normalization of gene expression in melanoma
Source: Sci Rep. 2022 Feb 28;12:3331. doi: 10.1038/s41598-022-07257-6 (PMC8885735; doi:10.1038/s41598-022-07257-6)
Supplement: Supplementary file 1 — Supplementary Information. [file 41598_2022_7257_MOESM1_ESM.pdf]

Innovative GenExpA software for selecting suitable reference genes for reliable normalization of gene expression in melanoma

Dorota Hoja-Łukowicz[1], Dawid Maciążek[2], Piotr Kościelniak[3], Marcelina E. Janik[1]

[1] Institute of Zoology and Biomedical Research, Jagiellonian University, Gronostajowa 9, 30-387 Krakow, Poland,

[2] Smoluchowski Institute of Physics, Jagiellonian University, Łojasiewicza 11, 30-348 Krakow, Poland

[3] Institute of Mathematics, Jagiellonian University, Łojasiewicza 6, 30-348 Kraków, Poland

Supplementary Data. NanoDrop spectrophotometer data of RNA purity. The number in parentheses refers to the biological replicate.

sample	RNA Conc. [ng/μl]	A260	A280	260/280	spectra
HEMa-LP (1)	162.6	4.065	1.925	2.11	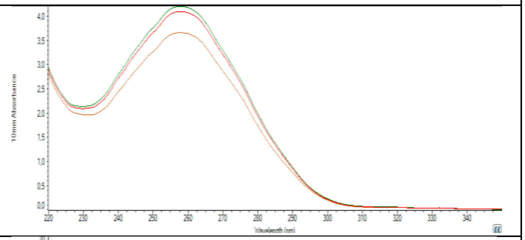
HEMa-LP (2)	166.8	4.169	1.984	2.10	
HEMa-LP (3)	145.2	3.630	1.726	2.10	
Mel202 (1)	582	14.551	6.973	2.09	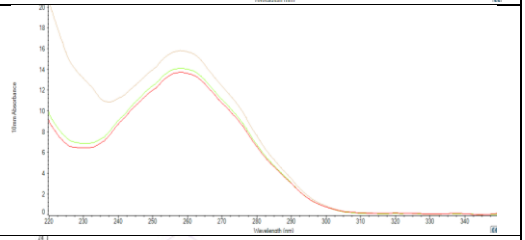
Mel202 (2)	680.7	17.017	7.966	2.14	
Mel202 (3)	586.2	14.656	7.014	2.09	
WM35 (1)	695.8	21.085	11.375	1.85	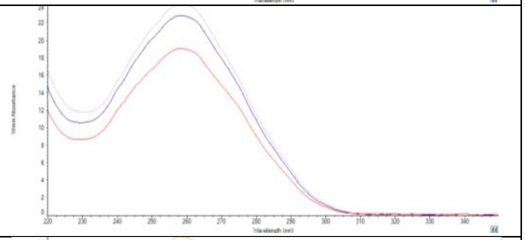
WM35 (2)	729.3	22.101	11.874	1.86	
WM35 (3)	930.1	28.185	15.223	1.85	
WM793 (1)	544.8	13.619	6.556	2.08	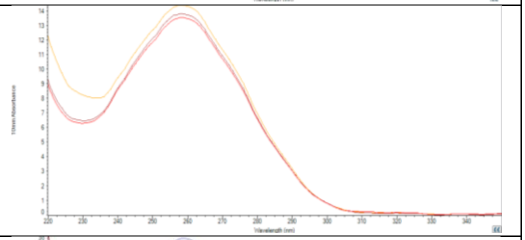
WM793 (2)	569.7	14.242	6.812	2.09	
WM793 (3)	590.6	14.766	7.103	2.08	
WM266-4 (1)	835.4	20.886	9.953	2.10	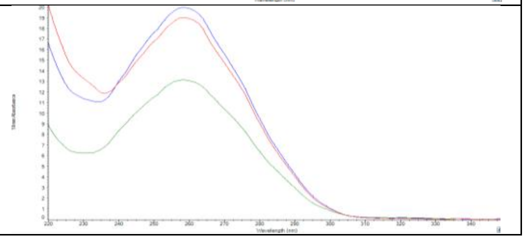
WM266-4 (2)	543.8	13.595	6.524	2.08	
WM266-4 (3)	782.2	19.554	9.326	2.10	
